# Study of the Chemical Composition and Biologically Active Properties of *Glycyrrhiza glabra* Extracts

**DOI:** 10.3390/life12111772

**Published:** 2022-11-02

**Authors:** Olga Babich, Svetlana Ivanova, Elena Ulrikh, Alexander Popov, Viktoria Larina, Andrej Frolov, Alexander Prosekov

**Affiliations:** 1 Research and Educational Center “Industrial Biotechnologies”, Immanuel Kant Baltic Federal University, A. Nevskogo Street 14, 236016 Kaliningrad, Russia; 2 Natural Nutraceutical Biotesting Laboratory, Kemerovo State University, Krasnaya Street 6, 650043 Kemerovo, Russia; 3 Department of General Mathematics and Informatics, Kemerovo State University, Krasnaya Street 6, 650043 Kemerovo, Russia; 4 Institute of Agroengineering and Food System, Kaliningrad State Technical University, Soviet Avenue 1, 236022 Kaliningrad, Russia; 5 K.A. Timiryazev Institute of Plant Physiology RAS, Botanicheskaya Uliza 35, 127276 Moscow, Russia; 6 Laboratory of Biocatalysis, Kemerovo State University, Krasnaya Street 6, 650043 Kemerovo, Russia

**Keywords:** *Glycyrrhiza glabra*, Soxhlet methanol extraction, chemical composition, biologically active substances, antibacterial, antioxidant activity

## Abstract

*Glycyrrhiza glabra* or licorice has long been known as a commonly used Ayurvedic herb. This study aims to investigate the effect of extraction methods on the chemical composition and biologically active properties of *Glycyrrhiza glabra* extract samples. The highest yield of the *Glycyrrhiza glabra* extract (21.31 ± 0.64 wt.%) was produced using the Soxhlet extraction method with methanol. The highest concentrations of biologically active substances (3,4-dihydroxybenzoic acid, n-coumaric acid, luteolin-7-glucoside, acacetin, apigenin-7-O-glucoside, chicoric acid, and hesperetin) were found in these samples of *Glycyrrhiza glabra* extracts. When applying the maceration method using a mixture of solvents methanol-NaOH, rosmarinic acid was identified, and catechin was found in large quantities with a mixture of methanol-trifluoroacetic acid (TFA). Growth inhibition zones were determined for *Escherichia coli* (13.6 ± 0.41 mm), *Pseudomonas aeruginosa* (10.8 ± 0.32 mm), *Bacillus subtilis* (16.1 ± 0.48 mm), and *Candida albicans* (13.2 ± 0.39 mm) when exposed to samples of *Glycyrrhiza glabra* extracts obtained by the Soxhlet method with methanol. The antioxidant activity of *Glycyrrhiza glabra* extract samples obtained by the Soxhlet method was 117.62 ± 7.91 µmol Trolox equivalent/g, using the ABTS method (highest value), and 23.91 ± 1.12 µmol Trolox equivalent/g according to the FRAP method (smallest). The antioxidant activity of the extract samples according to the DPPH method was an intermediate value of 58.16 ± 3.90 µmol Trolox equivalent/g. Antibacterial and antioxidant activities are manifested by the polyphenolic compounds and flavonoids contained in the samples of the methanol extract of *Glycyrrhiza glabra* produced using the Soxhlet method. These *Glycyrrhiza glabra* extract samples have the potential to become a natural alternative to existing therapies for the elimination of bacterial infections or the prevention of premature aging caused by free radicals and oxidative stress in the human body.

## 1. Introduction

Nature has always been a source of medicinal substances, providing man with a wide range of medicinal plants containing valuable phytochemicals. Licorice (*Glycyrrhiza glabra*) belongs to the bean family, and *Fabaceae*. *G. glabra* is a medicinal plant found throughout Asia, as well as in some parts of Europe [1]. It is believed that *G. glabra* originated in Iraq [2]. *G. glabra*, the most widespread species, is found in Italy, Spain, Turkey, the Caucasus, western China, and Central Asia [3]. *G. glabra* is one of the world’s most commercially valuable plants, with numerous applications in cosmetic, food, and pharmaceutical industries [4,5]. It is commercially grown in Italy, Spain, Greece, France, Iran, Iraq, Turkey, Turkmenistan, Uzbekistan, Syria, Afghanistan, Azerbaijan, India, China, USA, and England [4]. The plant can be a healthy food product and a natural sweetener because it contains herbal medicines with “food homology” [6]. One of about thirty licorice species is one of the most widely used components in feed and food formulations [7,8,9]. It is known that *G. glabra* contains biologically active substances (BASs) such as amino acids, proteins, simple sugars, polysaccharides, mineral salts, pectin, starches, sterols, gums, and resins [8].

According to the World Health Organization, *G. glabra* is used as a calming agent for sore throats and as an expectorant for catarrhal bronchitis [10,11,12,13,14]. *G. glabra* flavonoids are found in stem and root extracts and have demonstrated significant biological activity [15,16]. The main ones are four flavonoids of *G. glabra* (isoliquiritigenin, liquiritigenin, lihalocone, and glabridin), which have pharmacological activities. *G. glabra* has the potential to be a natural alternative to existing therapies for the treatment of new emerging diseases, with only minor side effects [4]. *G. glabra* contains glycyrrhizin, glycyrrhizic acid, amd isoliquiritin [17]. This plant can be used to treat dementia, cognitive impairment, and Alzheimer’s disease [18]. *G. glabra* roots, extracts, and active ingredients such as isoflavones, flavonoids, and glycyrrhizic acid have been shown to be effective in respiratory regulation, immunoregulation, antitumor, anti-inflammatory, gastrointestinal, and hepatoprotective activities [19,20], making it an important herbal medicine plant.

Textile dyeing is one of the applications for *G. glabra*. In the study [21], woolen fabrics were dyed using the roots of *G. glabra* L. The study aimed at giving dyed fabrics natural antibacterial properties. In the dyeing process, copper (II) sulfate, tin chloride, iron (II) sulfate, zinc chloride, and potassium-aluminum sulfate (alum) were used as mordants. Additionally, dyeing experiments were conducted without the use of a mordant, and dyed fabric samples were washed and dried at room temperature. After dyeing, the color efficiency of the dyed fabrics was measured, the staining structure was studied by scanning electron microscopy, FT-IR microspectroscopy, inductively coupled plasma mass spectrometry analyses, and antibacterial activity tests, and lightfastness and washing fastness tests were also carried out. These processes revealed that the roots of *G. glabra* L. can be used to dye woolen fabrics. Furthermore, stained tissue samples were found to have a good antibacterial effect against both Gram-positive (*Staphylococcus aureus*) and Gram-negative (*Escherichia coli*) bacteria [21].

Various methods for the extraction of biologically active substances from *G. glabra* are described in the literature [22,23,24,25]. The study [22] focused on the extraction of glycyrrhizic acid (GA) from *G. glabra* L. by a highly efficient and inexpensive extraction method. Water was chosen as the suitable solvent for the extraction process in order to eliminate the need for harmful and toxic solvents, conduct an environmentally friendly process, and reduce costs. The effect of various process parameters (extraction time, ratio of raw materials and solvent, pH of the extraction medium, and temperature) on the extraction and yield of extracts was studied. It was found that all four process parameters significantly affected the yield of extracts, and only the interaction between pH and extraction time was found to be important in increasing the % GA in the sediment. The highest yield was achieved experimentally, 54.9%, at an extraction time of 17 h, a raw material to solvent ratio of 10 g/mL, pH = 10, and at a temperature of 60 °C [22].

The study [23] showed that natural deep eutectic solvents (NADESs) are of growing interest in the scientific community as an alternative to conventional organic solvents. They consist exclusively of naturally occurring, non-toxic, environmentally friendly compounds. In this study, NADESe based on choline chloride and carboxylic acids are presented as new and green extraction media for GA extraction from *G. glabra*. Initial solvent screening indicated that the highest yield of extracts was achieved using a mixture of choline chloride and lactic acid at a 1:1 molar ratio. Various extraction conditions were investigated, such as 30% water in choline chloride solution: NADES lactic acid, liquid/solid ratio 40:1 (v/mL/g), temperature 40 °C, stirring speed 400 rpm, and extraction time 30 min. The yield of GA under these conditions was 53.72 ± 0.57 mg/g GA. The NADES extraction method successfully provided increased yield and improved anti-inflammatory activity of the extracts in vitro, which was evaluated using the human erythrocyte membrane stabilization method. In addition, it was found to be energy efficient and economical compared to traditional extraction methods. The results provided valuable information for the introduction of NADES to develop an extraction process for biologically active compounds of *G. glabra* [23].

A study [24] described supercritical fluid extraction (SFE) using a modified supercritical carbon dioxide co-solvent and a two-step separation/purification method, which was used as a way to increase the purity of glabridin, one of the many bioactive components of *G. glabra* (licorice). The SFE parameters were optimized using an analytical scale SFE system in the temperature range of 40–80 °C and pressure range of 10–50 MPa. The extraction was then increased 100-fold using the SFE preparative system under the following set of recommended conditions: 40 °C, 30 MPa, and SCCO_2_ modified with 25% (*v*/*v*) ethanol equivalent. The purity of glabridin obtained by the enhanced SFE system was 6.2%, which is much higher than the purity obtained by organic solvent extraction. The licorice extract obtained by scaling up the SFE system was isolated and purified using alcohol precipitation/filtration and adsorption chromatography with 80% aqueous ethanol to obtain a purer product. It was confirmed that the purity of glabridin in the final extract product increased to 37% without a significant loss of glabridin after two separation/purification steps [24].

The study [25] aimed to extract glycyrrhizic acid from *G. glabra*, which is widely used in medicine as an anti-inflammatory, antiulcer, antiallergic, and antipsoriatic agent. This study focused on extracting glycyrrhizic acid from the roots of *G. glabra* and assessing its anti-inflammatory activity in vitro. GA was extracted by maceration. Its physico-chemical properties, biochemical tests, and phytochemical properties were evaluated. Anti-inflammatory activity in vitro was assessed by the method of albumin denaturation. The results of the studies showed that the ash content and the yield of the extract were below the limits set by the Ayurvedic Pharmacopoeia of India. Flavonoids, saponins, and triterpenoids were identified in the *G. glabra* extract by phytochemical parameters. Thin layer chromatography showed a retention value of 0.5 cm. Percent inhibition indicated that *G. glabra* extract had some potential for healing inflammation. Thus, it was demonstrated that GA was successfully extracted from licorice roots. The evaluation parameters showed the presence of fewer impurities in the extract with the potential for anti-inflammatory action [25].

This study aims to investigate the effect of the extraction method on the chemical composition and biologically active properties of samples of *Glycyrrhiza glabra* extracts. The novelty of the research lies in the selection of the extraction (maceration) method, in which the maximum amount of BAS is released from *G. glabra*, exhibiting the most pronounced antimicrobial and antioxidant activities.

A null hypothesis was proposed prior to the start of the experiment, stating that the extraction method had no significant effect on the content of biologically active substances in *G. glabra* extracts, as well as their antioxidant and antibacterial activities. All extraction methods were assumed to be equally effective. 

## 2. Results

### 2.1. Total Yield of G. glabra Extracts

Table 1 presents the total yield of various *G. glabra* extracts.

The Soxhlet extraction method with methanol produced the highest yield of extracts; therefore, chromatograms for samples of *G. glabra* extracts obtained by the Soxhlet method with methanol as an extractant are provided below.

### 2.2. Analysis of BAS in G. glabra Extracts

**Figure 1:**
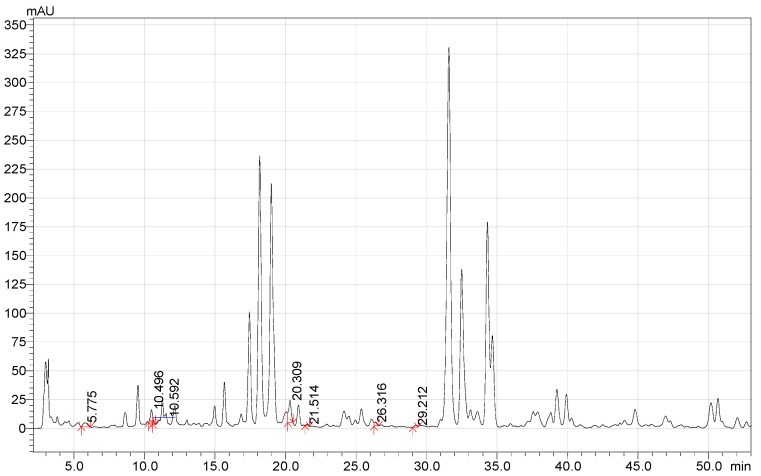
HPLC chromatogram of the *G. glabra* methanol extract (Soxhlet method).

**Figure 2:**
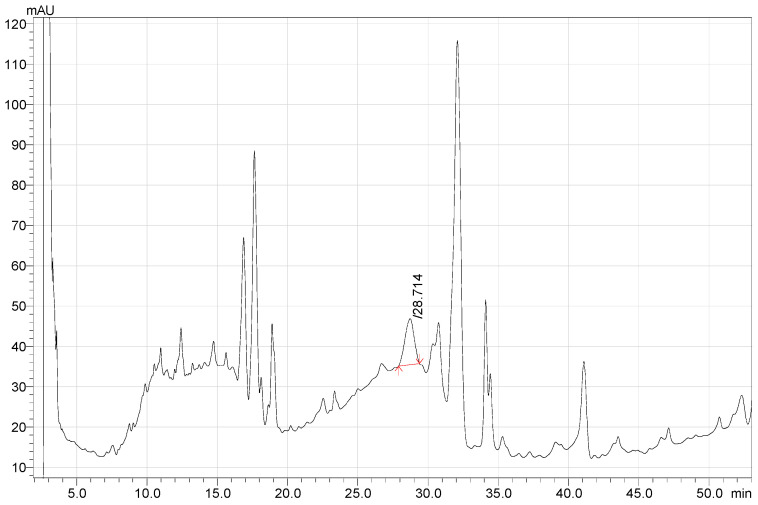
HPLC chromatogram of Methanol-NaOH extract of *G. glabra* (maceration method).

**Figure 3:**
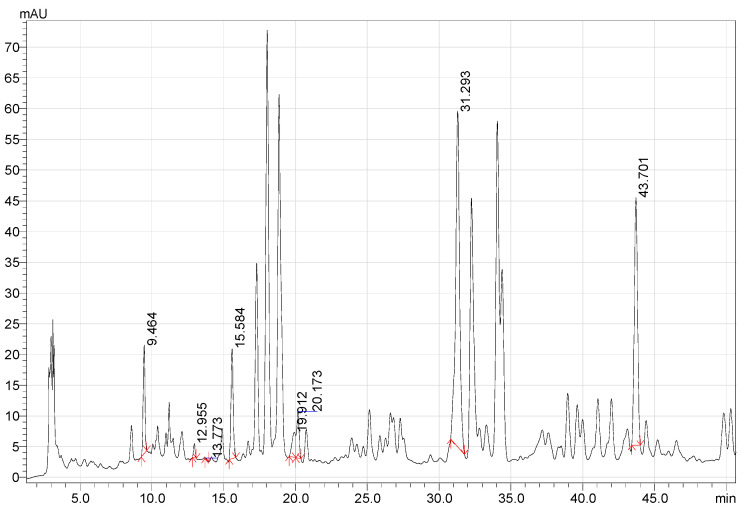
HPLC chromatogram of Methanol-TFA extract of *G. glabra* (maceration method).

Figure 1, Figure 2 and Figure 3 present the results of quantitative analysis of BAS in *G. glabra* extracts obtained using the Soxhlet method and maceration.

Table 2 presents the BAS present in various samples of *G. glabra* extracts analyzed by HPCL.

**Figure 4:**
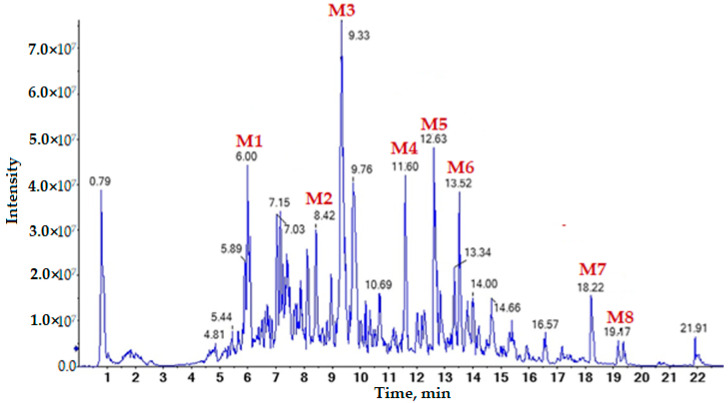
Chromatograms of the total ion current of the methanol extract of *G. glabra*, obtained by Soxhlet extraction, in the mode of the registration of negatively charged ions. The chromatographic signals of the secondary metabolites selected for MS/MS structure analysis were noted: 3.0 × 10^7^.

**Figure 5:**
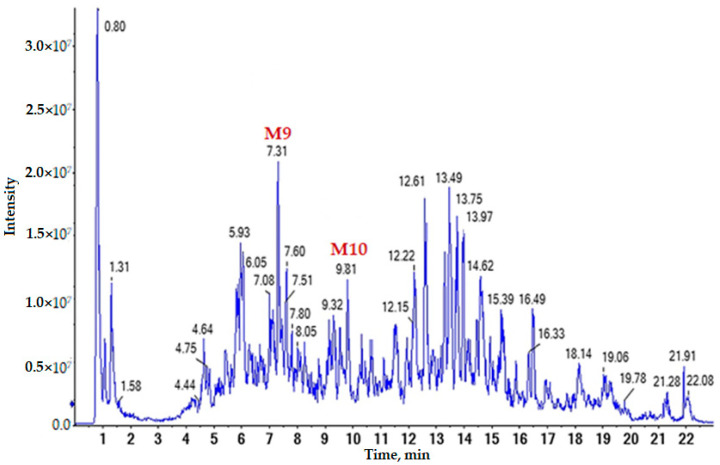
Chromatograms of the total ion current of the methanol extract of *G. glabra*, obtained by Soxhlet extraction, in the mode of the registration of positively charged ions. The chromatographic signals of the secondary metabolites selected for MS/MS structure analysis were noted.

Liquiritin apioside (Figure 4, peak M1), *G. glabra* G2 saponin (Figure 4, peak M2), glycyrrhizin (Figure 4, peak M3), lycoflavon A dimers (Figure 4, peak M4), and licoflavone derivative (Figure 4, peak M5); glabrol or hispaglabridin dimer (Figure 4, peak M6); dimers of linoleic (Figure 4, peak M7) and palmitic acids (Figure 4, peak M8); ononin dimer (Figure 5, peak M9); and formononetin (Figure 5, peak M10) were identified in the extract of the medicinal plant *G. glabra* obtained by the Soxhlet extraction method. The amount of these substances is presented in Table 3.

The fractionation of Soxhlet *G. glabra* extracts by HPLC identified an apigenin derivative with a yield of 1 × 10^2^ mg/kg at a retention time of 15.495 min.

### 2.3. Antibacterial Activity of G. glabra Extracts

Table 4 presents the antimicrobial activity of *G. glabra* extracts obtained by Soxhlet method and maceration methods with solvent mixtures. The antimicrobial activity of samples of *G. glabra* extracts, obtained by the Soxhlet method and maceration methods with solvent mixtures, was determined by the zone of inhibition of the growth of test microorganisms (diameter of the zone of inhibition of growth of test microorganisms) under the influence of these extracts. The diameter of the zone of inhibition of test microorganisms is presented in Table 4.

Dilutions of 2 and 5 times were prepared for extracts of *G. glabra* obtained using the Soxhlet method that demonstrated activities against these microorganisms in order to determine the minimum inhibitory concentration. However, none of the diluted extracts of *G. glabra* demonstrated activity against *P. aeruginosa*, *C. albicans*, *E. coli*, and *B. subtilis*.

### 2.4. Antioxidant Activity of G. glabra Extracts According to Soxhlet Method and Maceration Methods

Table 5 presents the results of the study of the antioxidant activity of *G. glabra* extracts according to Soxhlet and extracts obtained by maceration with a mixture of solvents methanol-NaOH and methanol-TFA.

## 3. Discussion

A comparison of data on the content of biologically active substances in samples of methanolic *G. glabra* extracts obtained by the Soxhlet method and presented in Table 2 shows that, in the *G. glabra* extract using pure methanol as an extractant, the largest amount of biologically active substances was identified: 3,4-dihydroxybenzoic acid, *n*-coumaric acid, luteolin-7-glucoside, acacetin, apigenin-7-O-glucoside, chicoric acid, and hesperetin. A significant amount of rosmarinic acid was identified when the maceration method was used with a mixture of solvents methanol-NaOH. A record amount of catechin was found when using the maceration method with a methanol-TFA mixture of solvents.

When studying the antibacterial activity, it was found that samples of the *G. glabra* extract obtained by Soxhlet extraction with methanol showed antimicrobial activity against all test strains of microorganisms. It is assumed that the presence of antimicrobial activity of this extract is due to the presence in its composition of such BAS as polyphenolic compounds and flavonoids: 3,4-dihydroxybenzoic acid, *n*-coumaric acid, luteolin-7-glucoside, acacetin, apigenin-7-O-glucoside, chicoric acid, and hesperetin. Saponins, alkaloids, and flavonoids contained in *G. glabra* extracts provide certain antibacterial activities. *G. glabra* preparations inhibit the activity of the bacterial genes of pathogenic microorganisms and slow down the growth of bacteria and their production of toxins that are harmful to the human body [26]. Hesperelin and a number of flavonoids contained in *G. glabra* have powerful antioxidant properties and thus protect the human body from the damaging effects of free radicals [27].

Soxhlet extraction is one of the most popular, simple, and effective extraction methods [28]. It is used for a wide range of samples such as soils, sediments, and animal and plant tissues. A wide range of organic solvents can be used for this method. The use of non-polar solvents alone is not recommended. Since its discovery in 1879, the standard Soxhlet method has been used regularly in almost every analytical laboratory. The Soxhlet extraction method continues to be the standard method against which the performance of modern extraction methods is compared. Over the years, intensive research has been carried out in various modifications to overcome the main shortcomings of the traditional Soxhlet method. New approaches to this method have been implemented to reduce extraction times and extractant volumes [29].

Recently, many studies have been devoted to modifications that include automated Soxhlet extraction, focused microwave Soxhlet extraction, ultrasonic Soxhlet extraction, high pressure Soxhlet extraction, and fluidized bed extraction. However, in all modifications, the Soxhlet extraction method remains the most accurate method with a high yield of extracts of various, including medicinal, plants [28].

We obtained the highest yield of extracts and biologically active substances from *G. glabra* using Soxhlet extraction with an organic solvent: methanol. In pharmaceutical technology, water, organic solvents and their mixtures, as well as aqueous solutions of acids and alkalis, can be used as selective solvents (extractants) during Soxhlet extraction [30].

The efficiency of the extraction process is characterized by the ability of the extracted substance to penetrate due to diffusion into the immobile solvent medium. The efficiency of diffusion and, consequently, of extraction increases with increasing temperatures and decreases with an increase in the viscosity of the medium and the size of the diffusing BAS particles. However, literature data show [28,30] that the fulfillment of the conditions of the diffusion law in some cases leads to a deterioration in the quality of the obtained extract [29,31]. Namely, the improper selection of the extractant, elevated temperature, and too fine grinding of raw materials leads to an increase in the proportion of ballast substances in the extract. The purification of such an extract requires additional technological stages [32]. When using methanol as an extractant in our studies, BAS from *G. glabra* pass to the full extent into extracts, and increasing the temperature when using the Soxhlet method also leads to a more complete yield of extractable BAS from *G. glabra*. As a result of the study, it was discovered that using methanol as an extractant in Soxhlet extraction results in a more complete extraction of biologically active substances from *G. glabra* while retaining significant antioxidant and antibacterial properties [29].

Samples of methanol extracts of *G. glabra* obtained by the Soxhlet method had the highest antibacterial activity (the largest diameter of the zone of inhibition of growth of all test microorganisms): 13.6 mm zone of inhibition of *E. coli*, 10.8 mm zone of inhibition of *P. aeruginosa*, 16.1 mm zone of inhibition of *B. subtilis*, and fungicidal activity: 13.2 mm zone of inhibition of *C. albicansa* (Table 4). For samples of *G. glabra* extracts obtained by the methanol-NaOH maceration method, the growth inhibition zone for *E. coli* was 6.7 mm, and the growth inhibition zone for *B. subtilis* was 8.6 mm. These extracts had no inhibitory effect on *P. aeruginosa* and *C. albicansa* strains. The growth inhibition zones for *P. aeruginosa, B. subtilis,* and *C. albicansa* for samples of *G. glabra* extracts obtained by the methanol-TFA maceration method were 6.4 mm, 10.2 mm, and 6.6 mm, respectively. These extracts did not have an inhibitory effect on E. coli strain.

When studying the antioxidant activity of *G. glabra* extracts obtained by the Soxhlet method and the biologically active derivative of apigenin isolated from the extracts by HPLC, presented in Table 5, it can be concluded that the *G. glabra* extracts have significant antioxidant activities. The highest antioxidant activity was found in the Soxhlet *G. glabra* extract samples using the ABTS method at 117.62 ± 7.91 µmol Trolox equivalent/g, while the lowest was found using the FRAP method: 23.91 ± 1.12 µmol Trolox equivalent/g.

The study demonstrated that the null hypothesis could not be confirmed. A significant effect of the extraction method on the content of biologically active substances in samples of *G. glabra* extracts, as well as on their antioxidant and antibacterial activities, was established.

The study [33] described the antimicrobial activity of *G. glabra*. The antimicrobial activity of the aqueous and methanol extracts of *G. glabra* leaves was analyzed against *Klebsiella pneumoniae*, *Candida albicans*, *Escherichia coli*, *Pseudomonas aeruginosa,* and *Enterococcus faecalis*. The results confirmed that the alcoholic extract of *G. glabra* had antimicrobial potential against *C. albicans* and Gram-positive bacteria in a dose-dependent manner. An alcoholic extract of *G. glabra* leaves has been shown to be effective against Gram-positive bacteria, suggesting that it could be a potential antimicrobial agent against a variety of bacterial strains [34].

Karahan et al. investigated the antioxidant and antimicrobial properties of methanolic extracts of *G. glabra* roots. MIC and disk diffusion methods were used to study antimicrobial efficacy. Antimicrobial analyses led to the conclusion that methanolic extracts of *G. glabra* roots are less effective against Gram-negative bacteria than against Gram-positive bacteria. In addition, methanolic extracts of *G. glabra* root were found to be more effective against *Candida* species than against other microorganisms. The results of the study showed that environmental factors affect the chemical content and biological properties of *G. glabra* in each habitat. In addition, the results of the study support the traditional practice of using *G. glabra* and suggest that it may be useful in the treatment of various infections [35]. Gupta et al. demonstrated that *G. glabra* roots have antimicrobial potential at a concentration of 500 g/mL in their study. They proved that the glabridin contained in the roots inhibits the growth and development of the Mycobacterium tuberculosis strain at a concentration of 29.16 µg/mL [36]. Research by Shirazi et al. also proved the antimicrobial properties of *G. glabra*. Studies showed that *G. glabra* inhibits the growth and development of *S. aureus* and *P. aeruginosa* test strains [37]. Thus, *G. glabra* has antimicrobial activity of both Gram-positive and Gram-negative bacteria [36,37,38].

*G. glabra* has practical applications due to its antioxidant properties, which are due to the presence of flavonoids, isoflavonoids, and other compounds [39,40]. Singh et al. [40] reported that compounds such as ispaglabridin A, glabridin, and 30-hydroxy-4-O-methylglabridin have high antioxidant activity. Later, it was proved that dihydrostilbene derivatives and lycochalcones B and D present in *G. glabra* can slow down oxidative processes [41,42]. In vitro studies, for example, demonstrated that licochalcones B and D can inhibit microsomal lipid peroxidation [42]. Thus, this proves the conclusion of Castangia et al. [43] who showed that *G. glabra* extract can be used in the production of innovative products for skin and cosmetics since it counteracts oxidative stress, maintaining skin homeostasis due to its high content of antioxidants.

Multidrug-resistant microorganisms are a serious problem in clinical medicine, and they require the search for new active substances. Various authors reported antimicrobial properties of *G. glabra*, especially against Gram-positive and Gram-negative bacteria such as *S. aureus*, *E. coli, P. aeruginosa, C. albicans*, and *B. subtilis*. [36,44]. Secondary metabolites, such as saponins, alkaloids, and flavonoids, are responsible for the observed antibacterial activity [8]. This activity is primarily attributed to glabridin, glabrol, glabren, hispaglabridin A, hispaglabridin B, 40-methylglabridin, and 3-hydroxyglabrol isolated from *G. glabra* [8]. The mechanism underlying this might be a decrease in bacterial gene expression, the inhibition of bacterial growth, and a reduction in bacterial toxin production [34]. In 2014, Ahn et al. [45] demonstrated that *G. glabra* prevents bacterial caries caused by *Streptococcus mutans* and *Streptococcus sobrinus*. Similarly, in vitro studies revealed that *G. glabra* aqueous and alcoholic extracts have inhibitory activity against *Streptococcus pyogenes* [34]. On the other hand, various authors have also reported the ability to inactivate methicillin-resistant *S. aureus* by downregulating SaeR and Hla, key virulence genes in *S. aureus* [34,45]. It is also assumed that lycochalcone E can be used for the chemical synthesis of new anti-*S. aureus* compounds that reduce α-toxin production in methicillin-sensitive *S. aureus* [34].

In addition, α-hemolysin is an important exotoxin in the pathogenesis of *S. aureus* infections [46]. Such infections are associated with a wide range of diseases, ranging from endocarditis to mild skin infections, toxinosis, and fatal pneumonia. Liquiritigenin, one of the most active compounds in *G. glabra*, was shown to prevent human lung cells (A549) from α-hemolysin-mediated damage by reducing α-hemolysin production [34,45] Similarly, glabrin and glycyrrhetinic acid demonstrated antibacterial activity against *S. aureus* [40].

Various authors reported the antibacterial activity of *G. glabra* against *M. tuberculosis* [34], demonstrating that glabridin rather than hispaglabridin B is responsible for this activity [14]. Antituberculous phenolic compounds have previously been identified in extracts of *G. glabra*, such as lycoisoflavone and lycochalcone A [47].

In a mouse lung infection model, *G. glabra* exhibited therapeutic activities against a multidrug-resistant strain of *P. aeruginosa* [48], and the hydroalcoholic extract led to a decrease in the microbial load in the blood, mainly due to the presence of stigmasterol, ergosterol, licochalcone, and glabridin [47].

*G. glabra* was also reported to be active against *Helicobacter pylori* [49]. According to Krausse et al. [49], the compounds glabridin and glabrene are responsible for this activity. Cao et al. [50] also reported that 18β-glycyrrhetinic acid significantly attenuated *H. pylori* gastritis infection. Asha et al. [51] found that flavonoid glabridin was active against *H. pylori*, while glycyrrhizin was inactive even at a concentration of 250 µg/mL. These flavonoids also showed activity against strains of *H. pylori* resistant to clarithromycin and amoxicillin [52]. The likely mechanism of this action is the inhibition of protein, DNA gyrase, and dihydrofolate reductase synthesis [52]. In addition, *G. glabra* polysaccharides also exhibit activity against the adhesion of *Porphyromonas gingivalis*, which is of great importance, since no specific adhesion inhibitors were described [53].

The antifungal activity of *G. glabra* is also described [54]. Sato et al. [54] reported that the methanol extract of *G. glabra* exhibits fungicidal activity against *Arthrinium sacchari* and *Chaetomium funicola*, while glabridin was found to be the active compound responsible for the observed effects [54]. In fact, isoflavones such as glabridin, glabrol, and their derivatives, are responsible for the in vivo inhibition of *M. smegmatis*, *Shigella*, *Salmonella*, *E. coli*, *S. mutans*, and *Lactobacillus acidophilus* [55]. Recently, Chandra and Gunasekaran [56] also demonstrated the antifungal activity of a crude methanol extract of *G. glabra* against *Aspergillus niger*.

Various authors reported that *C. albicans* is sensitive to *G. glabra* extracts due to their rich content of lykyritigenin, lykyritin, licochalcon A, and glabridin [57,58,59]. However, according to Karahan, Avsar, Ozyigit, and Berber [35], chemical content and biological and antimicrobial activities can be influenced by environmental conditions.

## 4. Materials and Methods

### 4.1. Reagents

All chemicals (ethylacetate, methanol, trifluoroacetic acid, formic acid, trichloroacetic acid, sodium hydroxide, and ultrapure water) used in the studies had at least ACS classification and were purchased from Sigma Aldrich (Sigma-Aldrich Rus, Moscow, Russia). Distilled water from a distiller (Khimlabpribor, Klin, Moscow, Russia) was used to prepare working solutions.

### 4.2. G. glabra Raw Materials for Extraction

*G. glabra* (licorice) samples were collected between June and August 2021 in the Kaliningrad region. The species affiliation of the biomaterial was confirmed by A.V. Pungin, the head of the herbarium of the Institute of Living Systems of the I. Kant Baltic Federal University (Protocol No. 9/2021). *G. glabra* was dried in a LUCH LShS-02 drying cabinet (DV-Ekspert, Moscow, Russia) at 30–40 °C, after which it was ground in an IKA Tube Mill control laboratory mill (DV-Ekspert, Moscow, Russia) up to 5 mm. Mature plants (leaves) were used to analyze the chemical composition of the *G. glabra* samples.

### 4.3. G. glabra Extraction

In the first stage, extraction parameters were chosen in accordance with the Soxhlet method in order to analyze the content of secondary metabolites of a phenolic nature. To achieve this, extraction was conducted using various organic extractants and pH values (methanol, methanol in an acidic medium, and methanol in an alkaline medium). Trifluoroacetic (TFA) and formic acids were used to create an acidic environment, and sodium hydroxide and ammonia were used to create an alkaline environment. Extracts were obtained by extraction and maceration (fractional extraction). The maceration process basically involved adding an extractant (methanol) to the crushed *G. glabra* raw material, sealing the vessel, and infusing the mixture at a temperature of 15–20 °C for 7 days while occasionally shaking or stirring. After infusion, the extract was poured out, and the leftover material was squeezed out, washed with a little extractant, and then squeezed out once more. The squeezed extract was combined with the drained extract, and the combined extract was brought back to its original volume using the extractant. This procedure was repeated three times. As a result, plant extracts of *G. glabra* were obtained by using 6 different methods:

- Extraction with methanol according to the Soxhlet method;
- Extraction with methanol by maceration;
- Extraction with methanol in a medium of 0.1 N sodium hydroxide by maceration (pH = 12.5);
- Extraction with methanol in a medium of 0.1 N ammonia solution by maceration (pH = 10.9);
- Extraction with methanol in a medium of 0.1 N trifluoroacetic acid by maceration (pH = 1.2);
- Extraction with methanol in a medium of 0.1 N formic acid by maceration (pH = 3.2).

Extraction with methanol according to the Soxhlet method was carried out for 8 h (15 cycles) at the boiling temperature of the solvent. However, according to literature data, an increase in temperature can cause the destruction of thermolabile biologically active substances (BAS) [54]; therefore, for comparison, extraction by maceration was performed without heating. Extraction by maceration was carried out for 8 h at room temperature with constant stirring and an extraction modulus of 1:40. During the experiment, the maximum yield of dry extract was evaluated.

### 4.4. Determination of the Qualitative Composition of G. glabra Extracts by Mass Spectrometry

To study the qualitative composition of *G. glabra* extracts by liquid chromatography-mass spectrometry, samples of extracts obtained by maceration with methanol, methanolic ammonia solution, and methanolic formic acid solution were analyzed using reverse-phase quadrupole time-of-flight ultra-high-performance chromatography-mass spectrometry (RP-UHPLC-Q-TOF-MS/MS) [55]. This analysis was performed using a Waters ACQUITY UPLC I-Class UPLC System (Waters GmbH, Eschborn, Germany) ultra-high-performance chromatograph (UHPLC) connected online to an AB Sciex TripleTOF 6600 hybrid quadrupole-time-of-flight mass spectrometer (Q-TOF-MS) (AB Sciex, Darmstadt, Germany) with electrospray (ES) ionization. Before performing the analysis of semi-polar metabolites, an optimization procedure was performed using extracts of test samples during which the optimal injection volume of the analyzed extract for analysis was established, which did not cause an overload of the analytical column and the associated negative changes in the linearity of measurements and the accuracy of determining the mass-to-charge ratio (*m*/*z*). The strategy of non-targeted metabolomic analysis [56] was used, in which UHPLC-MS/MS was performed in the data-independent acquisition (DIA) mode. The PHA algorithm was based on SWATH (sequential window acquisition of all theoretical fragment ion spectra mass spectrometry) technology. Two UHPLC-MS/MS experiments were performed. At the same time, the chromato-mass-spectrometric analysis was repeated twice to ensure the detection in the mode of both positively and negatively charged ions of secondary metabolites, [M + X]^+^ and [M-H]^−^, respectively, where X denotes singly charged cations. The two sets of obtained chromato-mass-spectrometric data were processed separately from each other. The quality of chromatographic–mass-spectrometric data (chromatographic and mass-spectrometric resolution, symmetry of chromatographic peaks, and accuracy of mass and retention time) was evaluated using the PeakView program (AB Sciex Pte. Ltd., PeakView 2.2 Software version, 2014, Colorado Springs, CO, USA).

### 4.5. Quantitative Determination of the Content of BAS in Extracts of G. glabra by HPLC

HPLC was performed on an LC-20AB Shimadzu Prominence chromatograph (Shimadzu, Kyoto, Japan) with a binary pump; SPD-M20A diode array detector (Shimadzu, Kyoto, Japan); and Zorbax 300SB-C18 4.6 mm × 250 mm 5 µm column (Agilent, Santa Clara, CA, USA). The separation was performed at a temperature of 40 °C in the gradient elution mode. Mobile phase: eluent A–0.1% TFA in distilled water, B–acetonitrile. The sample volume was 5 μL. The flow rate was 1 mL/min, and analytical wavelengths were 254, 280, and 325 nm.

The components were identified from the spectra of individual retention time of standard substances. We used analytical standards for individual substances purchased from Sigma Aldrich (Sigma-Aldrich Rus, Moscow, Russia) apigenin-7-O-glucoside (CAS 578-74-5, 93.47%), acacetin (CAS 480-44-4, ≥95% (HPLC)), astragalin (kaempferol-3-glucoside) (kaempferol-3-glucoside, CAS 480-10-4, 92.5%), hyperoside (CAS 482-36-0, analytical standard), hes-peretin -7-O-α-L-rhamnopyranoside (CAS 66513-83-5, analytical standard), quercetin 3-D-glucoside (CAS 482-35-9, ≥90.0%), catechin ((+)-catechin, CAS 154-23-4, ≥99.0%), luteolin 7-O-glucoside (CAS 5373-11-5, ≥98.0%), p-coumaric acid (CAS 501-98-4, ≥98.0%), rosmarinic acid ( CAS 20283-92-5, 96.0%), rutin (rutin hydrate, CAS 207671-50-9, ≥98.0%), trans-ferulic acid (CAS 537-98-4, analytical standard), trans-caffeic acid (CAS 501-16-6, ≥98.0%), chlorogenic acid, (CAS 327-97-9, ≥95.0%), chicoric acid (CAS 6537-80-0, ≥95.0%), and 3,4-dihydroxybenzoic acid (CAS 99-50-3, ≥97.0%).

Calibration curves were plotted to determine the concentrations of the components. The error of the method did not exceed 3–7%.

### 4.6. Determination of the Antimicrobial Activity of G. glabra Extracts

Samples of *G. glabra* extracts obtained by maceration with 0.1 N methanolic formic acid (methanol-TFA) were tested for antimicrobial activity [60]. When obtaining extracts, 100 mL of solvent accounted for 2.5 g of plant material. Solvents were removed from the extracts using an IKA RV 8 V vacuum rotary evaporator (DV-Ekspert, Moscow, Russia). The extracts were then dried using a Triad freeze dryer (Labconco Corporation, Kansas City, Missouri, USA). Drying conditions: vacuum 0.037 mbar, coolant temperature minus 80 °C. Extracts obtained from 2.5 g of plant material were dissolved in 10.0 mL of a mixture of methanol and water (9:1, respectively). The methanol:water ratio of 9:1 was chosen based on previous research and data from the literature on the use of mixed extractants for BAS extraction from plants [61,62].

The antimicrobial activity of extract samples and their individual active components was studied using the disk diffusion method. Four strains of test microorganisms were used for the study: Gram-positive bacteria *Bacillus subtilis* and Gram-negative bacteria *Escherichia coli*, *Pseudomonas aeruginosa* on LB agar medium, andyeast-like fungi of the genus *Candida albicans* on Ringer’s agar medium. Test strains of microorganisms *E. coli*, *P. aeruginosa*, *B. subtilis*, and *C. albicans* were purchased from the National Bioresource Center of the All-Russian Collection of Industrial Microorganisms of the Kurchatov Institute Research Center (Moscow, Russia). These microorganisms are standard and frequently used test strains for determining the antibacterial and fungicidal activities of various preparations, including medicinal plant extracts. These test strains of microorganisms were used in accordance with the recommendations of the National Committee for Clinical Laboratory Standards [63]. The growth inhibition of these microorganism test strains allows for the highly accurate determination of the *G. glabra* extract’s antibiotic activities. Clinical isolates of these test strains were obtained after inoculation on culture media and were used to determine the antibacterial activity of *G. glabra* extracts. The concentration of the microbial suspension was 1.5 × 10^8^ CFU/mL. The disc diameter was 6 mm, and the thickness of the agar layer was 4.0 ± 0.5 mm. The antibiotic kanamycin at a concentration of 50 µg/disk (for bacteria) and fluconazole 500 µg/disk (for yeast-like fungi) were used as controls. The comparison was carried out with a mixture consisting of 1% TFA (31%) and acetonitrile (69%), in which the individual active components were dissolved. The measurements were taken three times. The mean value was taken as the measurement result [64].

### 4.7. Determination of the Antioxidant Activity of G. glabra Extracts

When determining the antioxidant activity by the DPPH method, 20 µL of the extract sample and a solution of an individual compound or a standard solution (trolox) were mixed with 300 µL of a freshly prepared 0.1 mM solution of 2,2-diphenyl-1-picrylhydrazyl. The mixture was incubated in the dark at room temperature for 30 min. The decrease in optical density compared to the control, consisting of a 0.1 mm solution of 2,2-diphenyl-1-picrylhydrazyl and the appropriate solvent used for extraction or for the isolation of fractions and individual compounds, was recorded at 515 nm [65].

When determining the antioxidant activity by the ABTS method, a solution of the ABTS radical was preliminarily prepared. The ABTS radical was generated by mixing aliquots of 7.0 mM ABTS solution and 2.45 mM potassium persulfate solution. The solution was kept for 16 h in a dark place at room temperature. To start the reaction, 300 µL of the ABTS^+^ radical cation solution was added to 20 µL of the *G. glabra* extract, a solution of a single compound, or a standard (trolox). The absorbance was measured at 734 nm after the mixture was incubated for 15 min at 37 °C in the dark. A sample with the ABTS reagent and the corresponding solvent used for extraction or for the isolation of fractions and individual compounds was used as a control [66].

To determine the restorative power of *G. glabra* extracts, a freshly prepared FRAP reagent was used, prepared by mixing 10 parts of 0.3 M acetate buffer (pH 3.6), one part of a 10 mM solution of 2.4.6-tripyridyl-s-triazine in 40 mM HCl, and one part of aqueous 20 mM iron chloride solutio FeCl_3_ × 6H_2_O. The reaction was started by mixing 300 µL of the FRAP reagent and 20 µL of the test extract, a solution of an individual compound, or a standard solution (trolox). The reaction time was 10 min at 37 °C in the dark. The dptical density was measured at 593 nm. As a control, a sample with the FRAP reagent and the corresponding solvent used for extraction or for the isolation of fractions and individual compounds was used.

When measuring antioxidant activity using DPPH, ABTS, and FRAP methods, solutions of Trolox (6-hydroxy-2.5.7.8-tetramethylchroman-2-carboxylic acid) of known concentrations were used as standard solutions. When analyzing extracts, the results of the analyzes were expressed in µmol Trolox equivalents per gram of dry plant weight (µmol Trolox equivalent/g); when analyzing fractions and/or individual compounds, antioxidant activity was expressed in mmol Trolox equivalents per gram of individual compound (mmol trolox equivalents/g). All spectrophotometric measurements were performed using a CLARIOstar microplate reader (BMG Labtech, Ortenberg, Germany).

### 4.8. Statistical Analysis

Each experiment was repeated three times, and data are expressed as means ± standard deviation. Data processing was carried out via the standard methods of mathematical statistics. For each sample, three repeated preparations and three repeated measurements were performed (*n* = 3). The data were subjected to analysis of variance (ANOVA) using Statistica 10.0 (StatSoft Inc., 2007, Tulsa, OK, USA). Post hoc analyses (Duncan’s test) were undertaken to identify samples that were significantly different from each other. The equality of the variances of the extracted samples was checked using the Levene test. Differences between means were considered significant when the confidence interval is smaller than 5% (*p* < 0.05).

## 5. Conclusions

The Soxhlet extraction method with methanol resulted in the highest yield of *G. glabra* extract. Samples of the *G. glabra* extract obtained using the Soxhlet method with methanol demonstrated significant antibacterial activity against all test strains. The presence of antimicrobial activity of this extract can apparently be explained by the presence in its composition of such BAS as polyphenolic compounds and flavonoids. The highest antioxidant activity was found in methanol samples of *G. glabra* extracts (Soxhlet method) using the ABTS method, while the lowest was found using the FRAP method.

As a result of the studies, it was found that Soxhlet extraction with methanol is much more effective for isolating BAS contained in *G. glabra* compared to maceration (with methanol and with a mixture of solvents methanol-TFA, methanol-NH_4_OH, methanol-NaOH, and methanol-CHOOH). *G. glabra* extracts obtained via Soxhlet methanol extraction may become natural alternatives to existing therapies for the elimination of bacterial infections or the prevention of early aging in the human body due to free radicals and oxidative stress. The conducted studies allow filling a gap in the studies of BAS from *G. glabra*, growing in the Kaliningrad region. The number of phytocomponents and their activity are influenced by the methods used to obtain extracts of *G. glabra*. In order to extract the most biologically active substances (the Soxhlet extraction method), a rational extractant, methanol, was selected. The antioxidant and antibacterial effects of *G. glabra* extract samples were demonstrated in the studies, opening up the possibility of using these extracts as antioxidant and antibacterial agents in cosmetology, pharmacology, medicine, and the development of specialized functional food products with pronounced biological properties for the prevention and treatment of oxidative stress in the human body, as an alternative to synthetic antibiotics.

## Figures and Tables

**Table 1:** Total yield* (wt. %) of various *G. glabra* extracts.

Extraction Method					
Soxhlet Method	Maceration Method				
Extractant					
Methanol	Methanol	Methanol-NaOH	Methanol-NH_4_OH	Methanol-TFA **	Methanol-HCOOH
21.31 ± 0.64a	13.35 ± 0.40b	16.19 ± 0.51b	15.84 ± 0.47b	18.41 ± 0.55ab	15.66 ± 0.47b

* extract yield; wt.%—extract yield; percentage by weight. ** TFA—trifluoroacetic acid. Values in row followed by the same letter do not differ significantly (*p* < 0.05) as assessed by the post hoc test (Duncan’s test). Data presented as a mean ± SD (*n* = 3).

**Table 2:** Content of phytochemicals (mg/kg) present in various samples of *G. glabra* extracts analyzed by HPCL.

BAS *	BAS Release Time, min	Extraction Method		
		Soxhlet	Maceration	
		Extractant		
		Methanol	Methanol-NaOH	Methanol-TFA
3,4-dihydroxybenzoic acid	5.8	3.68 ± 0.11	-	-
Caffeic acid	10.5	traces	-	-
Chlorogenic acid	10.6	traces	-	-
n-coumaric acid	13.8	1.00 ± 0.03	-	Below detection limit
Ferulic acid	16.6	traces	-	traces
Rutin	19.6	traces	-	-
Luteolin-7-glucoside	21.5	0.88 ± 0.03	-	-
Astragalin	25,3	traces	-	-
Rosmarinic acid	28.7	traces	53.21 ± 1.59	-
Acacetin	54.9	15.45 ± 0.46	-	traces
Apigenin-7-O-glucoside	26.3	2.10 ± 0.06	-	-
Chicoric acid	21.5	5.43 ± 0.16	-	traces
Catechin	9.6	traces	-	1468.43 ± 44.05
Hesperetin	48.0	1.75 ± 0.05	-	-
Daidzein	31.3	-	-	230.45 ± 6.91

* BAS—Biologically active substances.

**Table 3:** Mass spectrometric analysis of metabolites of Soxhlet *G. glabra* extracts using RP-UHPLC-CFP-MS/MS.

Peaks	tR ^1^, min	Ion Type	Molecular Ion Mass (*m*/*z*), Experimental	Molecular Ion Mass (*m*/*z*), Calculated	Mass Accuracy, ppm ^2^	Chemical Formula	Fragmentation	Identification
M1	6	[M−H]-	1099.3402 */549.17	1099.33 */549.1614	9.20	C_52_H_60_O_26_ */ C_26_H_30_O_13_	417.1217, 255.0676	liquiritin apioside
M2	8.4	[M−H]-	837.3984	837.3914	8.84	C_42_H_62_O_17_	-	*G. glabra* G2 saponin
M3	9.3	[M−H]-	821.401	821.3965	5.48	C_42_H_62_O_16_	351.0578	glycyrrhizin
M4	11.6	[M−H]-	643.2335	643.2337	0.31	C_40_H_36_O_8_	321.113	icoflavon A dimer l
M5	12.6	[M−H]-	647.2715	647.265	10.00	C_40_H_40_O_8_	323.1311	licoflavone derivative dimer
M6	13.5	[M−H]-	783.3919	783.3902	2.17	C_50_H_56_O_8_	391.1904, 203.0739, 187.1152	glabrol dimer or hispaglabridin dimer
M7	18.2	[M−H]-	559.4728	559.4732	0.71	C_36_H_64_O_4_	279.2328	linoleic acid dimer
M8	19.2	[M−H]-	511.4706	511.4732	5.08	C_32_H_64_O_4_	255.2318	palmitic acid dimer
M9	7.3	[M+H]+	861.2611	861.26	1.28	C_44_H_46_O_18_	431.1351, 269.0812	ononin dimer
M10	9.8	[M+H]+	269.0809	269.0808	0.37	C_16_H_14_O_4_	-	formononetin

* dimer; ^1^ tR, min—retention time, min; ^2^ mass accuracy, ppm—mass accuracy, parts per million.

**Table 4:** Growth inhibition zones of test microorganisms of *G. glabra* extracts obtained by the Soxhlet method and maceration methods with solvent mixtures.

Extract	Diameters of Growth Inhibition Zones of Test Microorganisms, mm			
	*E. coli*	*P. aeruginosa*	*B. subtilis*	*C. albicans*
Soxhlet extraction with methanol	13.6 ± 0.41a	10.8 ± 0.32a	16.1 ± 0.48a	13.2 ± 0.39a
Methanol-NaOH maceration	6.7 ± 0.20b	-	8.6 ± 0.26b	-
Methanol-TFA maceration	-	6.4 ± 0.19b	10.2 ± 0.26b	6.6 ± 0.26b
Kanamycin 50 µg	17.3 ± 0.52c	12.7 ± 0.38a	28.4 ± 0.85c	–
Fluconazole 500 µg	–	–	–	20.5 ± 0.62c

Values in columns followed by the same letter do not differ significantly (*p* < 0.05) as assessed by the post hoc test (Duncan’s test). Data presented as a mean ± SD (*n* = 3).

**Table 5:** Antioxidant activity of *G. glabra* extracts obtained using the Soxhlet method and maceration.

Extracts	Antioxidant Activity, µmol * Trolox Equivalent/g		
	ABTS ^1^	DPPH ^2^	FRAP ^3^
Methanol (Soxhlet)	117.62 ± 7.91 a	58.16 ± 3.90 a	23.91 ± 1.12 a
Methanol-NaOH maceration	12.08 ± 0.62 b	2.42 ± 0.13 b	0.031 ± 0.002 b
Methanol-TFA maceration	13.53 ± 0.41 b	11.84 ± 0.36 c	1.09 ± 0.03 b

* µmol—micromol; ^1^ ABTS—2,2′-azino-bis(3-ethylbenzothiazoline-6-sulfonic acid); ^2^ DPPH—2,2-diphenyl-1-picrylhydrazyl; ^3^ FRAP—ferric reducing antioxidant power. Values in columns followed by the same letter do not differ significantly (*p* < 0.05) as assessed by the post hoc test (Duncan’s test). Data presented as a mean ± SD (*n* = 3).

## Data Availability

Data are contained within the article.

## References

[1] Fiore C., Eisenhut M., Ragazzi E., Zanchin G., Armanini D. (2005). A history of the therapeutic use of liquorice in Europe. J. Ethnopharmacol..

[2] Mamedov N.A., Egamberdieva D. (2019). Phytochemical Constituents and Pharmacological Effects of Licorice: A Review. Plant Hum. Health.

[3] Hayashi H., Yokoshima K., Chiba R., Fujii I., Fattokhov I., Saidov M. (2019). Field survey of Glycyrrhiza plants in Central Asia (5). Chemical characterization of G. bucharica Collected in Tajikistan. Chem. Pharm. Bull..

[4] Wahab S., Annadurai S., Abullais S.S., Das G., Ahmad W., Ahmad M.F., Kandasamy G., Vasudevan R., Ali M.S., Amir M. (2021). *Glycyrrhiza glabra* (Licorice): A Comprehensive Review on Its Phytochemistry, Biological Activities, Clinical Evidence and Toxicology. Plants.

[5] Jiang M., Zhao S., Yang S., Lin X., He X., Wei X., Song Q., Li R., Fu C., Zhang J. (2020). An “Essential Herbal Medicine”—Licorice: A Review of Phytochemicals and Its Effects in Combination Preparations. J. Ethnopharmacol..

[6] Esmaeili H., Karami A., Hadian J., Nejad E.S., Otto L.G. (2020). Genetic structure and variation in Iranian licorice (*Glycyrrhiza glabra* L.) populations based on morphological, phytochemical and simple sequence repeats markers. Ind. Crop. Prod..

[7] Pastorino G., Cornara L., Soares S., Rodrigues F., Oliveira M.B.P.P. (2018). Liquorice (*Glycyrrhiza glabra*): A phytochemical and pharmacological review. Phytother. Res..

[8] Wang L., Yang R., Yuan B., Liu Y., Liu C. (2015). The antiviral and antimicrobial activities of licorice, a widely-used Chinese herb. Acta Pharm. Sin. B.

[9] Rizzato G., Scalabrin E., Radaelli M., Capodaglio G., Piccolo O. (2017). A new exploration of licorice metabolome. Food Chem..

[10] Xiaoying W., Han Z., Yu W. (2017). Glycyrrhiza glabra (Licorice). Sustained Energy for Enhanced Human Functions and Activity.

[11] Alsayari A., Wahab S. (2021). Genus Ziziphus for the treatment of chronic inflammatory diseases. Saudi J. Biol. Sci..

[12] Bao F., Bai H.Y., Wu Z.R., Yang Z.G. (2021). Phenolic compounds from cultivated *Glycyrrhiza uralensis* and their PD-1/PD-L1 inhibitory activities. Nat. Prod. Res..

[13] El-Saber Batiha G., Magdy Beshbishy A., El-Mleeh A., Abdel-Daim M.M., Prasad Devkota H. (2020). Traditional uses, bioactive chemical constituents, and pharmacological and toxicological activities of *Glycyrrhiza glabra* L. (Fabaceae). Biomolecules.

[14] Richard S.A. (2021). Exploring the Pivotal Immunomodulatory and Anti-Inflammatory Potentials of Glycyrrhizic and Glycyrrhetinic Acids. Mediat. Inflamm..

[15] Thakur A.K., Raj P. (2017). Pharmacological perspective of *Glycyrrhiza glabra* Linn.: A Mini-Review. J. Anal. Pharm. Res..

[16] Graebin C.S., Mérillon J.-M., Ramawat K.G. (2018). The Pharmacological Activities of Glycyrrhizinic Acid (“Glycyrrhizin”) and Glycyrrhetinic Acid. Sweeteners, Reference Series in Phytochemistry.

[17] Ahmed-Farid O.A., Haredy S.A., Niazy R.M., Linhardt R.J., Warda M. (2019). Dose-dependent neuroprotective effect of oriental phyto-derived glycyrrhizin on experimental neuroterminal norepinephrine depletion in a rat brain model. Chem.-Biol. Interact..

[18] Sharma S., Chourasia R., Pandey A., Rai A., Sahoo D., Belwal T., Nabavi S.F. (2021). Alzheimer’s disease: Ethanobotanical studies. Naturally Occurring Chemicals Against Alzheimer’s Disease.

[19] Wahab S., Ahmad I., Irfan S., Siddiqua A., Usmani S., Ahmad M.P. (2022). Pharmacological Efficacy and Safety of *Glycyrrhiza glabra* in the treatment of respiratory tract infections. Mini-Rev. Med. Chem..

[20] Akram M., Nawaz A. (2017). Effects of medicinal plants on Alzheimer’s disease and memory deficits. Neural Regen. Res..

[21] Fazlıhan Y. (2020). Application of *Glycyrrhiza glabra* L. Root as a Natural Antibacterial Agent in Finishing of Textile. Ind. Crops Prod..

[22] Khanahmadi M., Ghaffarzadegan R., Khalighi-Sigaroodi F., Naghdi Badi H., Mehrafarin A., Hajiaghaee R. (2018). Optimization of the Glycyrrhizic Acid Extraction from Licorice by Response Surface Methodology. IJCCE.

[23] Lanjekar K.J., Rathod V.K. (2021). Green extraction of Glycyrrhizic acid from *Glycyrrhiza glabra* using choline chloride based natural deep eutectic solvents (NADESs). Process. Biochem..

[24] Hong J.H., Jung I.I., Cho Y.K., Haam S., Lee S.-Y., Lim G., Ryu J.-H. (2019). Preparation of High-quality Glabridin Extract from *Glycyrrhiza glabra*. Biotechnol. Bioprocess E.

[25] Chauhan S., Gulati N., Nagaich U. (2018). Glycyrrhizic acid: Extraction, screening and evaluation of anti-inflammatory property. Ars Pharm..

[26] Helmenstine A.M. (2021). Null Hypothesis Definition and Examples. ThoughtCo..

[27] Zverev Y.F. (2017). Flavonoids through the eyes of a pharmacologist. Antioxidant and anti-inflammatory activity. Rev. Clin. Pharmacol. Drug Ther..

[28] Kapadia P., Newell A.S., Cunningham J., Roberts M.R., Hardy J.G. (2022). Extraction of High-Value Chemicals from Plants for Technical and Medical Applications. Int. J. Mol. Sci..

[29] Zygler A., Słomińska M., Namieśnik J., Pawliszyn J. (2012). 2.04-Soxhlet Extraction and New Developments Such as Soxtec.

[30] Abduraxmanov B., Mamatkhanova M., Satimov G., Khalilov R., Mamatkhanov A. (2018). Study of the process of extraction of the sum of flavonoids from the aerial part of *Glycyrrhiza glabra*. Process. Devices Model..

[31] Mamatkhanova M.A., Abduraxmanov B., Nigmatullaev B.A., Satimov G., Khalilov R., Mamatkhanov A.U. (2016). Studying the aboveground part of *Glycyrrhiza glabra* as a perspective raw material for the production of preparations based on flavonoids. Chem. Plant Raw Mater..

[32] Abduraxmanov B., Satimov G., Khalilov R., Mamatkhanov A. (2021). Technology for obtaining a substance based on flavonoids of the aerial part of *Glycyrrhiza glabra*. Pharm. Chem. J..

[33] Atefi D., Turgay Erdo Ö. (2003). Antimicrobial activities of various medicinal and commercial plant extracts T›bbi ve Ticari Amaçl› Kullan›lan Baz› Bitki Ekstraktlar›n›n Antimikrobiyal Etkileri. Turk. J. Biol..

[34] Irani M., Sarmadi M., Bernard F., Ebrahimi G.H., Bazarnov H.S. (2010). Leaves antimicrobial activity of *Glycyrrhiza glabra* L. Iran. J. Pharm. Res..

[35] Karahan F., Avsar C., Ozyigit I.I., Berber I. (2016). Antimicrobial and antioxidant activities of medicinal plant *Glycyrrhiza glabra* var. glandulifera from different habitats. Biotechnol. Biotechnol. Equip..

[36] Gupta V.K., Fatima A., Faridi U., Negi A.S., Shanker K., Kumar J.K., Rahuja N., Luqman S., Sisodia B.S., Saikia D. (2008). Antimicrobial potential of *Glycyrrhiza glabra* roots. J. Ethnopharmacol..

[37] Shirazi M.H., Ranjbar R., Eshraghi S., Sadeghi G., Jonaidi N., Bazzaz N., Izadi M., Sadeghifard N. (2007). An Evaluation of antibacterial activity of *Glycyrrhiza glabra* Extract on the growth of *Salmonella*, *Shigella* and ETEC *E. coli*. J. Biol. Sci..

[38] Chen K., Yang R., Shen F.-Q., Zhu H.-L. (2019). Advances in Pharmacological Activities and Mechanisms of Glycyrrhizic Acid. Curr. Med. Chem..

[39] Varsha A.R., Sonam P. (2013). Phytochemical screening and determination of anti-bacterial and anti-oxidant potential of *Glycyrrhiza glabra* root extracts. J. Environ. Dev..

[40] Singh V., Pal A., Darokar M.P. (2015). A polyphenolic flavonoid glabridin: Oxidative stress response in multidrug-resistant Staphylococcus aureus. Free Radic. Biol. Med..

[41] Biondi D.M., Rocco C., Ruberto G. (2003). New dihydrostilbene derivatives from the leaves of *Glycyrrhiza glabra* and evaluation of their antioxidant activity. J. Nat. Prod..

[42] Sharma V., Katiyar A., Agrawal R.C., Mérillon J.-M., Ramawat K.G. (2016). Glycyrrhiza glabra: Chemistry and Pharmacological Activity. Sweeteners, Reference Series in Phytochemistry.

[43] Castangia I., Caddeo C., Manca M.L., Casu L., Latorre A.C., Diez S.O., Manconi M. (2015). Delivery of liquorice extract by liposomes and hyalurosomes to protect the skin against oxidative stress injuries. Carbohydr. Polym..

[44] Wang Q., Qian Y., Wang Q., Yang Y.-F., Ji S., Song W., Ye M. (2015). Metabolites identification of bioactive licorice compounds in rats. J. Pharm. Biomed. Anal..

[45] Ahn S.-J., Song Y.-D., Mah S.-J., Cho E.-J., Kook J.-K. (2014). Determination of optimal concentration of deglycyrrhizinated licorice root extract for preventing dental caries using a bacterial model system. J. Dent. Sci..

[46] Berube B.J., Bubeck Wardenburg J. (2013). Staphylococcus aureus α-toxin: Nearly a century of intrigue. Toxins.

[47] Chakotiya A.S., Tanwar A., Srivastava P., Narula A., Sharma R.K. (2017). Effect of aquo-alchoholic extract of *Glycyrrhiza glabra* against Pseudomonas aeruginosa in mice lung infection model. Biomed. Pharmacother..

[48] Chakotiya A.S., Tanwar A., Narula A., Sharma R.K. (2016). Alternative to antibiotics against Pseudomonas aeruginosa: Effects of *Glycyrrhiza glabra* on membrane permeability and inhibition of efflux activity and biofilm formation in Pseudomonas aeruginosa and its in vitro time-kill activity. Microb. Pathog..

[49] Krausse R., Bielenberg J., Blaschek W., Ullmann U. (2004). In vitro anti-Helicobacter pylori activity of extractum liquiritiae, glycyrrhizin and its metabolites. J. Antimicrob. Chemotech..

[50] Cao D., Jiang J., You L., Jia Z., Tsukamoto T., Cai H., Cao X. (2016). The protective effects of 18β-glycyrrhetinic acid on Helicobacter pylori-infected gastric mucosa in Mongolian gerbils. BioMed Res. Int..

[51] Asha M.K., Debraj D., Prashanth D., Edwin J.R., Srikanth H.S., Muruganantham N., Agarwal A. (2013). In vitro anti-Helicobacter pylori activity of a flavonoid rich extract of *Glycyrrhiza glabra* and its probable mechanisms of action. J. Ethnopharmacol..

[52] Fukai T., Marumo A., Kaitou K., Kanda T., Terada S., Nomura T. (2002). Anti-Helicobacter pylori flavonoids from licorice extract. Life Sci..

[53] Leite C.d.S., Bonafé G.A., Carvalho Santos J., Martinez C.A.R., Ortega M.M., Ribeiro M.L. (2022). The Anti-Inflammatory Properties of Licorice (*Glycyrrhiza glabra*)-Derived Compounds in Intestinal Disorders. Int. J. Mol. Sci..

[54] Sato J., Goto K., Nanjo F., Kawai S., Murata K. (2000). Antifungal activity of plant extracts against Arthrinium sacchari and Chaetomium funicola. JBB.

[55] Ajagannanavar S.L., Battur H., Shamarao S., Sivakumar V., Patil P.U., Shanavas P. (2014). Effect of aqueous and alcoholic licorice (*Glycyrrhiza glabra*) root extract against Streptococcus mutans and Lactobacillus acidophilus in comparison to chlorhexidine: An in vitro study. Int. J. Oral Health Dent..

[56] Chandra J.H., Gunasekaran H. (2017). Screening of the phytochemical, antimicrobial and antioxidant activity of *Glycyrrhiza glabra* root extract. J. Environ. Biol..

[57] Vazquez-Morado L.E., Robles-Zepeda R.E., Ochoa-Leyva A., Arvizu-Flores A.A., Garibay-Escobar A., Castillo-Yañez F., Lopez-Zavala A.A. (2021). Biochemical characterization and inhibition of thermolabile hemolysin from Vibrio parahaemolyticus by phenolic compounds. PeerJ.

[58] Lee J.B., Jeong Y.A., Ahn D.J., Bang I.S. (2019). SPME-GC/MS Analysis of Methanol in Biospecimen by Derivatization with Pyran Compound. Molecules.

[59] Xujun R., Wang Y., Zhou L., Zheng Q., Hao H., He D. (2022). Evaluation of Untargeted Metabolomic Strategy for the Discovery of Biomarker of Breast Cancer Frontiers. Pharmacology.

[60] Gonelimali F.D., Lin J., Miao W., Xuan J., Charles F., Chen M., Hatab S.R. (2018). Antimicrobial Properties and Mechanism of Action of Some Plant Extracts Against Food Pathogens and Spoilage Microorganisms. Front. Microbiol..

[61] Plant Material Used, Extraction and Isolation of Natural Compounds-PLOS. https://journals.plos.org.

[62] Truong D.-H., Nguyen D.H., Ta N.T.A., Bui A.V., Do T.H., Nguyen H.C. (2019). Evaluation of the Use of Different Solvents for Phytochemical Constituents, Antioxidants, and In Vitro Anti-Inflammatory Activities of *Severinia buxifolia*. J. Food Qual..

[63] Barry A.L. (1999). National Committee for Clinical Laboratory Standards. Methods for Determining Bactericidal Activity of Antimicrobial Agents: Approved Guideline.

[64] Mostafa A.A., Al-Askar A.A., Almaary K.S., Dawoud T.M., Sholkamy E.N., Bakri M.M. (2018). Antimicrobial activity of some plant extracts against bacterial strains causing food poisoning diseases. Saudi J. Biol. Sci..

[65] Chaves N., Santiago A., Alías J.C. (2020). Quantification of the Antioxidant Activity of Plant Extracts: Analysis of Sensitivity and Hierarchization Based on the Method Used. Antioxidants.

[66] Oleinik G., Dario P.P., de Morais Gasperin K., Benvegnú D.M., Lima F.O., Soares L.C., Gallina A.L. (2022). *In vitro* antioxidant extracts evaluation from the residue of the *Hevea brasiliensis* seed. Sci. Rep..

